# The Sex and Gender Intersection in Chronic Periodontitis

**DOI:** 10.3389/fpubh.2017.00189

**Published:** 2017-08-04

**Authors:** Effie Ioannidou

**Affiliations:** ^1^Periodontology, UCONN Health, Farmington, CT, United States

**Keywords:** sex, gender, periodontitis, gender inequality, sex biology

## Abstract

Periodontitis, a complex polymicrobial inflammatory disease, is a public health burden affecting more than 100 million people and being partially responsible for tooth loss. Interestingly, periodontitis has a documented higher prevalence in men as compared to women signifying a possible sex/gender entanglement in the disease pathogenesis. Although relevant evidence has treated sex/gender in a simplistic dichotomous manner, periodontitis may represent a complex inflammatory disease model, in which sex biology may interfere with gender social and behavioral constructs affecting disease clinical phenotype. Even when it became clear that experimental oral health research needed to incorporate gender (and/or sex) framework in the hypothesis, researchers overwhelmingly ignored it unless the research question was directly related to reproductive system or sex-specific cancer. With the recognition of gender medicine as an independent field of research, this study challenged the current notion regarding sex/gender roles in periodontal disease. We aimed to develop the methodological and analytical framework with the recognition of sex/gender as important determinants of disease pathogenesis that require special attention. First, we aim to present relevant sex biologic evidence to understand the plausibility of the epidemiologic data. In periodontitis pathogenesis, sex dimorphism has been implicated in the disease etiology possibly affecting the bacterial component and the host immune response both in the innate and adaptive levels. With the clear distinction between sex and gender, gender oral health disparities have been explained by socioeconomic factors, cultural attitudes as well as access to preventive and regular care. Economic inequality and hardship for women have resulted in limited access to oral care. As a result, gender emerged as a complex socioeconomic and behavioral factor influencing oral health outcomes. Taken together, as disease phenotypic presentation is a multifactorial product of biology, behavior and the environment, sex dimorphism in immunity as well as gender socio-behavioral construct might play a role in the above model. Therefore, this paper will provide the conceptual framework and principles intergrading sex and gender within periodontal research in a complex biologic and socio-behavioral dimension.

## Introduction

Periodontitis, a complex polymicrobial inflammatory disease, affects more than 30% of the US population (approximately 100 million people) (1) and is partially responsible for full edentulism in 1/4 of US adults 65 years of age or older (2). Therefore, understanding the disease and determining the most effective therapy it is a priority, as highlighted by the 2020 Healthy People Objective (OH-5), which aims to reduce the number of adults with moderate or severe periodontitis.

Interestingly, periodontitis has a documented higher prevalence in men (~57%) compared to women (~39%) (1, 3), signifying a possible sex/gender bias in disease pathogenesis. Important contributing disease factors, such as diabetes and smoking, do not seem to significantly differ between genders, as the prevalence of diabetes is 9.8% in men and 9.2% in women (4), whereas the prevalence of smoking is 18.8% in men and 14.8% in women. Furthermore, classic studies of the natural history of periodontal diseases have been conducted as single-gender studies focusing only on men (5–12). Hence, their findings have limited validity for half of the world’s population and therefore questionable generalizability. Recent evidence on periodontal risk assessment has revealed that gender plays a critical role in periodontal risk (13). Specifically, when the analysis is limited to the severe periodontitis category, men are at higher risk compared to women (13). These data also confirmed the role of smoking and diabetes as contributory factors in the disease process (13).

Although relevant evidence has treated sex/gender in a simplistic dichotomous manner (14), periodontitis may by a complex inflammatory disease model, in which sex might interfere with the gender social construct, affecting the disease clinical phenotype and therapeutic response (14–16). Yet, gender bias has not previously been evaluated in periodontal trials.

As defined by the Institute of Medicine, sex is a biological variable that is determined by the chromosomal structure (male [XY] or female [XX]) as well as reproductive organs and functions assigned by chromosomal complement (17). However, gender is fluid, self-perceived, and determined by responses to social institutions as well as influenced by gender roles, social expectations, and sexual identity (18, 19).

A series of historical events, medical evidence, and political decisions influenced the attention on research hypotheses exploring sex/gender differences. Undeniably, following the atrocities of WWII as related to unethical medical experiments on prisoners in concentration camps, there was an urgent need for a regulatory framework for the protection of human subjects including women and children (17). As a result, women were not allowed to participate in clinical trials based on: (1) the general assumption of there being “no difference” between women and men and (2) the notion that including women in trials would confound study outcomes with unnecessary hormonal “noise” and fluctuations. As the understanding of the biologic implications of sex and socio-behavioral dimensions of gender evolved, it became apparent that certain population groups might require a separate focus. Consequently, in 1986, the NIH encouraged (but did not require) the inclusion of women in clinical studies (17). Unfortunately, within a few years, the NIH realized that the suggestion was not regularly implemented in clinical studies (20). Thus, after establishing the NIH Office of Research on Women’s Health, the Revitalization Act (P.L. 103-43) became law to ensure that women and minorities would be included in clinical research. A few years later, the NIH updated its guidelines, requiring a description of analytical plans by sex/gender for each study. In 2015, the NIH developed a policy on the consideration of sex as a biological variable, requiring studies to adopt the appropriate terms and to also justify single-sex research protocols (21).

With the current emphasis placed on sex/gender as determining factors in preclinical and clinical studies of health and disease by the NIH (21), European Commission (22), and Canadian Institutes of Health (23), medicine has taken a more critical look at existing evidence by investigating differences between sex/gender in the diagnosis, prevention, and treatment of several diseases. As a result, medical research focusing on sex/gender disparities has significantly increased in recent years (24). For example, a US Drug Safety report revealed that drugs that were withdrawn between 1997 and 2000 presented a greater risk for women than men (25), highlighting the gender bias in required drug trial designs. Quality analysis of medical trials has confirmed that a gender bias exists in many large clinical studies, such as the Physician’s Health Study on aspirin and Multiple Risk Factor Intervention Trial (MRFIT), which enrolled no women (26). In addition, the EU-funded project EUGenMed examined, in a multidisciplinary manner, the roadmap for the inclusion of gender aspects in European biomedical and health research (25) (EUGenMed 2015). Collectively, these efforts signify that the field of gendered medicine has recently evolved with the gradual increase of research publications, particularly in cardiology, since the 1990s (24).

Even when it became clear that experimental oral health research needed to incorporate the gender (and/or sex) framework in hypotheses, researchers overwhelmingly ignored this information unless the research question was related to the reproductive system or sex-specific cancer. With the recognition of gender medicine as an independent field of research, this paper challenges the current notion regarding the sex/gender role in chronic periodontitis pathogenesis (27), given the strong epidemiological evidence suggesting a difference in the prevalence of periodontitis between men and women, which remains constant in every disease stage and under various case definitions (1). This disparity has been attributed to gender behavioral factors, which received significant weight, with the goal to maintain the assumption of similar therapeutic responses between genders. In this process, several factors have been ignored or have not been extensively evaluated, including sex biology and gender behavior, which have been increasing recognized in complex chronic and/or immune disease models.

Given that sex and gender have been significantly understudied in the field of periodontology, we aimed to develop a methodological and analytical framework that recognizes sex/gender as important determinants of periodontitis with the goal to address gender bias in clinical as well as preclinical studies in periodontology.

### Sex Biology in Periodontitis: A Microbiome–Host Approach

Consistent epidemiological data have highlighted higher periodontitis prevalence in men as compared to women (28, 29). Given that periodontitis is a complex inflammatory disease of microbial etiology, at the microbial level, the sex hypothesis might be tangled with the immune-regulatory dimension. Although in infectious diseases, evolutionary evidence has consistently supported a male disadvantage in prevalence, outcomes, and survival rates (30), these findings could not be directly extrapolated to periodontitis due to its chronic inflammatory profile triggered by bacteria. Hence, the hormonal, genetic, and epigenetic influence on immunity has remained unclear and possibly understudied.

In periodontitis pathogenesis, sex dimorphism might be implicated in disease microbial etiology possibly modifying the bacterial biofilm, as well as the host immune response (27, 31). Indeed, limited data from earlier studies have shown significantly higher odds for harboring salivary and subgingival periodontal pathogens, such as *Prevotella intermedia*, in men than in women (32, 33). Similarly, sex-specific differences have also been observed in the gut microbiome (34), where *Bacteroides* have a lower abundance in women than in men. When fecal microbiota data were analyzed according to gender, higher levels of *Bacteroides* and *Prevotella* species were observed in men than in women (35). Following this direction, recent evidence has revealed a diverse interaction between microbes and host sex hormones (36) with microbes manipulating and utilizing sex hormones to survive. More specifically, in mouse models, there has been a direct shift in hormonal levels (i.e., production of androgens in female mice) after transferring gut commensal bacteria from male to female mice (37). Other groups have discovered sex-distinct signatures in the gut microbiome, which after being mediated by testosterone, could upregulate a certain immune response and affect the initiation and progression of diabetes type 1 in mouse models (38). In similar models, microbes could use sex steroids for growth as well as transmission and, therefore, explain the evolutionary process of microbial survival (36).

As the role of inflammation in periodontal pathogenesis has evolved, the host immune response has taken on an important part (39, 40). Therefore, sex biology could be explored as a modifying factor of innate and adaptive responses possibly manifesting a diverse susceptibility to the disease (15, 27, 31). Sex steroids and X-linked genes have been proposed to be the main mechanisms that alter immune function (41–43). Although periodontitis has primarily been associated with X-linked genetic disorders, the reported evidence is of low quality and not conclusive (44). Therefore, in this report, we aim to examine basic evidence regarding the sex influence on the immune response in genetic or autoimmune disease models.

Sex chromosomes play an important role in mediating the differences in the immune response, with X-linked genes regulating pattern recognition receptors and cytokine production, as well as transcriptional factors (41). The X chromosome’s significance in immunity was confirmed in inherited syndrome models (i.e., Klinefelter), in which the extra X chromosome in men resulted in an immune response similar to that in women (with a high CD4 T cell count, high CD4/CD8 ratio, and higher immunoglobulin levels) compared to XY men controls (45). On the contrary, studies on women with Turner syndrome (X monosomy) have shown lower T cell and B cell levels as well as low IgG and IgM levels, weak PMN chemotaxis, and low CD4/CD8 ratio as compared to women with chromosomal XX (43, 46). In parallel, studies have shown polymorphisms in sex chromosome genes that encode receptors for anti-inflammatory IL-4, IL-10, and IL-12 (30, 41, 43) indicating a sex bias in pro- and anti-inflammatory immune responses. Certain polymorphisms in sex chromosomal and autosomal genes have also been hypothesized to affect immune responses, including cytokine production, pattern recognition receptors, and transcriptional factors (15), and contribute to the differences between sexes. Additional lessons were learned from the autoimmune disease models, where a clear connection to the X chromosome might be implicated in the loss of the immune tolerance (47).

In addition, hormonal mediators of the immune response (i.e., estrogens, progesterone, and testosterone) have been shown to affect innate and adaptive immunity (48). In general, several studies have demonstrated the immunosuppressive role of testosterone and progesterone, as well as the immune-enhancing impact of estrogens (48), which collectively explain the high infection rate in males combined with the high autoimmune disease prevalence in female mammals (41, 43, 48, 49). In human autoimmune disease models, women represent more than 80% of cases (41). Interestingly, in animal autoimmune models, the incidence of autoimmune diseases is increased by male castration and decreased by female ovariectomy (50). Animal and human studies have revealed that increased estrogen levels lead to higher neutrophil numbers and enhanced phagocytosis (51). Furthermore, female macrophages exhibit higher levels of toll-like receptor 7 expression, with higher phagocytic activity (43), and produce more interferon a (TNF-a). An additional mechanism for sex dimorphic characteristics is involved in the function of TLR4, which in animal and *in vitro* models has shown greater expression in males than females, followed by increased production of pro-inflammatory cytokine, leading to a more pronounced inflammatory response (52).

Estrogens have demonstrated a bi-potential effect on the immune response, with low doses enhancing pro-inflammatory cytokine production and high or sustained doses reducing pro-inflammatory cytokine production (15, 53). Female animal models have also demonstrated an increased Th2 immune response, with the IL-4, IL-5, and IL-10 levels confirming the possible inhibition of disease progression (27), as well as increased proliferation of M2 macrophages amplifying the immune response (54). Although women have higher levels of T lymphocytes than men, their adaptive immunity is predominantly driven by B cells and CD4 Th2 cells (27). Although the increased antibody production in women determines the response to infections and vaccination, it also increases the risk for autoimmune diseases such as Sjogren’s syndrome, multiple sclerosis and others (41, 55). There are some indications that while Th2-mediated diseases tend to be more prevalent in women (43), Th1-mediated autoimmune diseases, such as cardiomyopathy, may be more prevalent in men (54). In men, experimental evidence has shown that testosterone increases monocyte production of IL-12 in response to LPS stimulation, with increased Th1 differentiation and NK cell activation (56, 57). In addition, regulatory T cells, which have anti-inflammatory properties, have been found at increased levels in men (30), although there have been some contradictory results in mouse studies. Although the sex-mediated immune pathways were not verified in periodontitis, these established concepts would need to be examined in animal and human models of periodontitis.

In fact, in an effort to apply the above basic principles to the periodontal hypothesis, limited clinical experimental data have supported a higher concentration of the IgG antibody against *Porphyromonas gingivalis* in women than men, similar to chronic periodontitis (58). In summary, given that the innate immune response might be more regulated in women (53) and more intense in men, women tend to be more effective at pathogen clearance compared to men. In addition, exposure of NK cells to estrogens increases INF-γ production (59), which has been shown to play a controversial role in periodontitis, with *in vivo* studies confirming an association with bone resorption and *in vitro* studies showing an inhibitory role in osteoclastogenesis (60, 61). At the final level of inflammation resolution, there are indications that women may produce higher levels of resolvins due to increased synthesis of long-chain n-3 PUFA, leading to more effective periodontal inflammation resolution (27).

Given the sex influences on microbial communities and immune functions, the host–microbial hypothesis in periodontal pathogenesis might need to be examined under the sex lens in order to achieve an unbiased understanding.

### Gender As a Socio-Behavioral Construct

Gender oral health disparities have been explained by socioeconomic factors and cultural attitudes, as well as by access to preventive and regular care (62). There is a clear distinction between the terms “sex” and “gender.” While sex refers to the biological factors that are directly related to genetics, physiology, and anatomy, including the reproductive system, gender relates to social roles, behaviors, and attitudes (63, 64). More importantly, gender identity, as a product of perception, social influence, and relations, has been frequently reduced to a binary measure (64), which tends to simplify complex social interactions. Therefore, although animal models may be able to capture sex differences, gender differences, as socio-behavioral processes, have been more difficult to capture (65).

In this context, efforts were made to define gender after considering several psychosocial variables that determine gender roles, gender identity, and relations, as well as social norms (65). Therefore, composite gender scores were developed to capture the dimension of the socio-behavioral constructs, to measure masculine and feminine personality characteristics and to assess the manner in which they might affect disease presence and outcomes (66). Using this methodology, the association between gender-related factors and biological sex was investigated, and seven gender-related variables, which were able to independently predict sex, were confirmed, including the household primary earner, income, and housework weekly hours, status of the primary person responsible for doing housework, stress levels at home, and Bem Sex-Role Inventory (65, 66). This methodology recognized the intersection between sex and gender, as well as their relationship with cardiovascular outcomes. In summary, femininity was associated with high levels of anxiety and depression, smoking, diabetes, hypertension, and poor cardiovascular outcomes. Interestingly, patients with feminine personality traits were less likely to be prescribed as antihypertensive and be prescribed statin medications.

Gender inequality has affected economic and health outcomes internationally for women and children (67, 68). Women experience a higher incidence and severity of poverty than men (“feminization of poverty”) (69, 70), as confirmed by the Institute for Women’s Policy Research (IWPR) gender poverty report (71). The International Monetary Fund recently demonstrated that income inequality is associated with gender inequality after controlling for confounders (72). Interestingly, this association is true for all countries, with advanced countries showing a stronger correlation between inequality and economic participation, while in low-income countries, inequality of the opportunity for education and political empowerment and health appear to be the most important barriers to income distribution (72). Ironically, although women represent half of the world’s workforce, they only generate 37% of the global gross domestic product (73).

Economic inequality has been a recognized determinant of health care that affects access to health care, including dental care (69, 74). Indeed, disparities resulting from economic inequality are greater in the US compared to other wealthy countries (67, 75, 76). Although the Affordable Care Act (ACA) aimed to expand coverage, 39% of below-average Americans still avoid seeing a doctor due to cost (75). In addition, fewer women are uninsured compared to men, but women with health insurance are responsible for higher out-of-pocket costs compared to men, regardless of their insurance type (Medicare or employer-sponsored) (75). The Agency for Health Care Research and Quality has produced data on disparities in health-care quality according to which women are significantly more likely than men to be delayed or unable to obtain medical and dental care or prescription medication (77). As timely care delivery has been shown to reduce mortality and morbidity (78), this finding is significant and could result in poor chronic disease outcomes. The disparities are more important when they intersect with race and ethnicity. Hispanic women experience a statistically significant delay in care compared to non-Hispanic women (77). Another important finding highlights the fact that 2% of women, compared to 0.9% of men, receive prescriptions for at least one medication that should be avoided due to adverse events (77). Based on National Health Medicaid data (1997–2011) (79), when assessing dental care access, approximately 20% of women, as opposed to 15% of men, “did not receive dental care due to cost” (79). In fact, economic inequality has resulted in only 38% of middle class Americans having annual dental visits compared to 55% of high-income Americans (80). Even among high-income Americans, income measurements were negatively but significantly correlated with the number of missing and decayed teeth only in women (80). Based on the above data, gender economic inequality has been found to directly affect access to care and tooth loss (80).

Despite the continuous increase of health-care costs, preventive care utilization has always been a goal for cost reduction. The factors that predict the dental care utilization rate include gender, high income, and overall health perception (74). In the pre-health reform era, women utilized preventive care more frequently than men, although still at low rates and often only for acute care (74). In addition, cultural norms influence men’s overall low primary care service utilization because masculinity drives the expectation for men to stay strong and to not need care (74).

After implementation of the ACA, followed by the expansion of adult Medicaid in 27 states, adult dental benefits increased by 2–6% points; however, the changes did not reach the level of significance (81). Specifically, it has been projected that by 2026 (assuming that the ACA remains in place), approximately 45% of the population will use dental preventive services, with an annual growth rate of 0.5% (81). With preventive services dominating the utilization model, treatment service will decline, following the demographic shifts in the population.

Risk perception has also been affected by gender, with different concern levels on disease risks or treatment decisions observed (62). As a result, attitude and behavior related to health promotion and compliance might be affected. In the same context, women have been consistently found to demonstrate better oral hygiene habits than men. Oral health-related behaviors in women, including brushing and flossing, have occurred at higher rates than in men (62). However, given all of the above data, the differences in oral hygiene might be a simplistic explanation for the differences in disease presentation between women and men.

In conclusion, gender emerges as a complex socioeconomic and behavioral complex factor that certainly affects access to care, treatment choices, and outcomes; therefore, it needs to be appropriately studied and analyzed.

## Considering Sex/Gender in Periodontal Research

Recent efforts by research organizations (NIH, FDA, and CIHR) (21–23, 82, 83), the International Committee of Medical Journal Editors (ICMJE) (84), the European Association of Science Editors (85, 86), and others (Gender Innovations, Stanford University) (63) have emphasized sex and gender and developed guidelines and checklists to address their intersection. Because of this international effort, oral health and periodontal research may need to establish a framework to produce diverse and generalizable knowledge, reduce health disparities, avoid gender bias, and improve oral health therapeutic interventions without any *a priori* assumptions (64, 87).

The above goal is realistic because the developed checklists aim to help researchers to develop their strategies and analytical plans, in terms of sex and gender, after they first determine the importance and relevance of sex/gender or the manner in which they might intersect (88). As emphasized above, sex remains a relevant factor in the preclinical research (89) setting with animal, tissue, and cell models, and must also be addressed at the preclinical level, as the NIH has recently required (21).

Furthermore, to facilitate extensive and reliable literature searches, search engine tools were developed with the goal of limiting biomedical searches to sex- and/or gender-related references in a predictable and complete manner (62, 90). Moreover, after analytical plans have been designed, there are certain recommendations for the presentation of study populations at baseline to reflect the representation of men and women, with special consideration given to age, ethnic/racial background, and socioeconomic status. It is important to have appropriate statistical methods to analyze sex/gender differences at baseline, as well as at the end of the intervention. Result disaggregation and reporting will enable the preclinical and clinical research community to evaluate treatments and better understand therapeutic options by sex/gender. When randomly reviewing periodontal randomized controlled trials, we found that, at baseline, the trial demographics were appropriately presented; however, the outcomes were never disaggregated in terms of gender (Ioannidou, unpublished data). This finding highlights the lack of evidence for periodontal treatment responses by gender.

In addition to the above data, journal editors and publishers may need to reinforce the ICMJE guidelines, which require appropriate use of the terms sex/gender in scientific publications, report of the sex/gender of participants, report of the sex of animals or cells, and discussion of the influence of sex/and or gender on the study findings (84, 85). These guidelines offer transparency in reporting but also provide an interpretation of results with an aim toward generalizability to the general population. The guidelines also emphasize that when studies are conducted on single-sex/gender populations, the reason should be justified and reflected in the study title to avoid misleading interpretations.

Given the role of sex and gender in chronic periodontitis pathogenesis, periodontal researchers need to “set it up” and extensively explore their role and effects on disease pathogenesis, clinical presentation, and therapy. Therefore, when considering future study designs, periodontal researchers must apply principles that allow for high levels of external validity that reduce sex/gender bias. For this purpose, researchers must overcome historical sex/gender assumptions, recognize the potential implications of sex/gender on the hypothesis, and address these variables appropriately in their study designs.

## Author Contributions

The author has conceived, designed, and conducted the study.

## Conflict of Interest Statement

The author declares that the research was conducted in the absence of any commercial or financial relationships that could be construed as a potential conflict of interest.
